# A 42-year-old patient with renal cell carcinoma presenting as low back pain: A case report

**DOI:** 10.1097/MD.0000000000037639

**Published:** 2024-03-29

**Authors:** Ga Yang Shim, Min-Su Kim, Hong Jun Kim, Yewan Park, So-Woon Kim, Myung Chul Yoo

**Affiliations:** a Department of Physical and Rehabilitation Medicine, College of Medicine, Kyung Hee University, Seoul, Korea; b Division of Medical Oncology-Hematology, Department of Medicine, College of Medicine, Kyung Hee University, Seoul, Korea; c Department of Internal Medicine, Kyung Hee University Medical Center, Seoul, Korea; d Department of Pathology, College of Medicine, Kyung Hee University, Seoul, Korea.

**Keywords:** atypical, back pain, neoadjuvant, renal cell carcinoma

## Abstract

**Rationale:**

Renal cell carcinoma (RCC) is the most common renal neoplasm, accounting for 2.4% of all cancers in Korea. Although the usual clinical manifestations of RCC include flank pain, hematuria, and palpable mass, RCC is generally characterized by a lack of early warning signs and is mostly discovered incidentally in advanced stage. This case report describes a 42-year-old Korean man diagnosed with giant RCC who presented with simple back pain.

**Patient concerns:**

The clinical manifestation of a 42-year-old Korean man was chronic back pain.

**Diagnoses:**

Contrast-enhanced computed tomography showed a 19.1-cm sized heterogeneous enhancing mass on the right kidney and tumor thrombosis extending into inferior vena cava.

**Intervention:**

Due to the large size of the tumor and extensive tumor thrombosis, the multidisciplinary team decided to administer neoadjuvant chemotherapy and an anticoagulant. Following 12 cycles of treatment with nivolumab and cabozantinib, he underwent a right radical nephrectomy with an adrenalectomy and tumor thrombectomy.

**Outcomes:**

Treatment was successful and posttreatment he started a cancer rehabilitation program. He was followed-up as an outpatient and no longer complains of back pain.

**Lessons:**

RCC can manifest clinically as back pain, with diagnosis being difficult without appropriate imaging modalities. RCC should be included in the differential diagnosis of patients with low back pain, even at a young age.

## 1. Introduction

Renal cell carcinoma (RCC) is the most common type of kidney cancer and the tenth leading cause of cancer in Korea, accounting for 2.4% of all cancers.^[1]^ Its incidence is 2 times higher in men than in women, with peak incidence in patients aged in their 60s and 70s.^[1,2]^ According to the Korea National Cancer Incidence Database, the incidence of RCC has increased over the past 30 years, but the survival rate has improved.^[1]^ The improvement in survival may be a result of earlier detection due to widespread use of radiological testing. Nevertheless, RCC remains a lethal malignancy, with a crude mortality rate expected to reach 2.1 per 100,000 individuals in 2023.^[3]^

The classic triad of RCC includes flank pain, hematuria, and a palpable abdominal mass. Most patients with RCC, however, are asymptomatic or exhibit nonspecific symptoms such as fatigue. Approximately half are identified incidentally on radiographic examination, with 30% of these tumors being in advanced stages, including locally invasive or metastatic disease, at the time of diagnosis.^[4,5]^ The mean RCC size at diagnosis has been reported to be 5.4 cm, with larger tumor size being associated with greater malignant potential.^[6]^ Giant RCC, defined as tumor volume > 1000 mL, is rare. This report describes a 42-year-old Korean man complaining of simple back pain who was diagnosed with a giant RCC.

## 2. Case description

A 42-year-old Korean man with low back pain received injection treatments at a pain clinic several times over 2 years, but showed no improvement. He subsequently visited a tertiary hospital for right lower back pain of an intensity of 3/10 on the Numerical Rating Scale, but without radiating pain. He had no relevant medical history, but had recently lost 5 kg in weight over 4 months accompanied by a loss of appetite. He had a family history of cancer on his father’s side, as his father had been diagnosed with colon cancer and his paternal grandmother diagnosed with uterine cancer. He was a current smoker with 15 pack-years of cigarette smoking, and consumed 2 or more drinks of alcohol per week. Physical examination showed that he had pale conjunctiva and a palpable, non-tender mass on the right upper quadrant of the abdomen, about 8 finger breadths in size.

**Figure 1.**
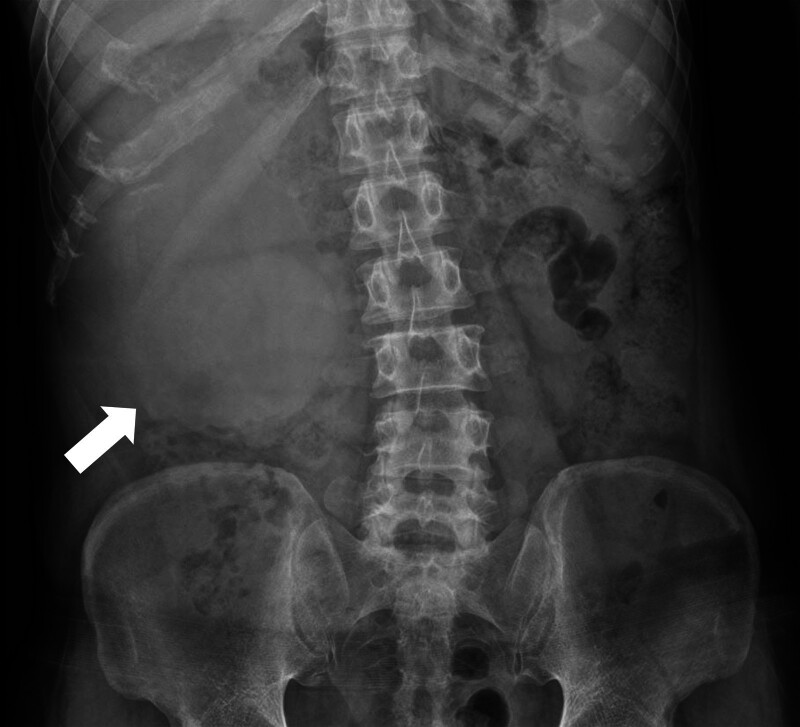
Abdominal radiography, anterior–posterior view, of this patient, showing a mass-like opacity (arrow) in the right abdomen.

**Figure 2.**
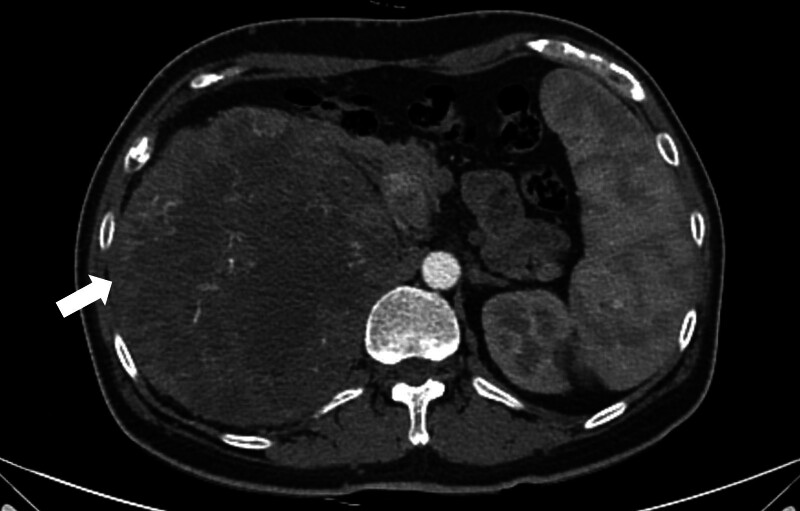
Abdominal computed tomography, axial view, of this patient, showing a heterogeneous enhancing mass (arrow) replacing the right kidney with nephromegaly (long diameter, 19.1 cm), with the mass extending into the renal pelvis and proximal ureter, suggesting a malignant renal tumor.

**Figure 3.**
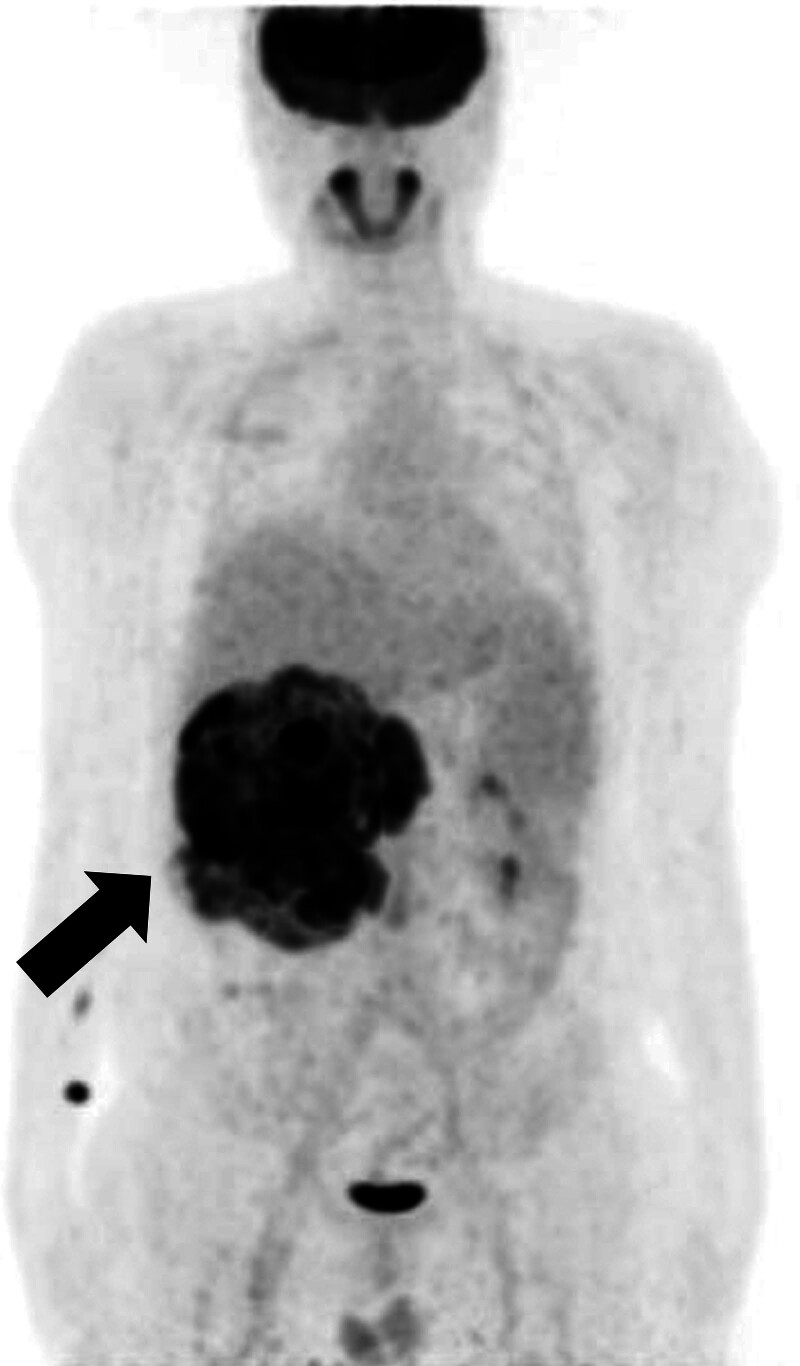
Whole-body positron emission tomography/computed tomography, anterior-posterior view, of this patient, showing a huge hypermetabolic lesion in the right kidney with maximum standardized uptake values of 13.3 (arrow), consistent with RCC, and involving the proximal ureter. Probable metastatic lymph nodes were detected in the retrocaval and aortocaval areas, along with tumor thrombosis in the right renal vein and inferior vena cava.

Abdominal radiography (Fig. 1) revealed a mass-like opacity in the right abdomen, suggesting a hidden tumor. Contrast-enhanced computed tomography showed a 19.1 cm sized heterogeneous enhancing mass on the right kidney extending into the renal pelvis and proximal ureter, along with tumor thrombosis extending into the right renal vein and inferior vena cava below the diaphragm. These findings suggested a malignant renal tumor, such as RCC, that corresponded to T3b or T4 on the American Joint Committee on Cancer TNM staging system, 8th edition (Fig. 2).^[7]^ Histological analysis of a specimen obtained by ultrasound-guided biopsy showed a clear cell RCC. Whole-body positron emission tomography/computed tomography, however, showed a huge hypermetabolic lesion throughout the right kidney, but no evidence of metastatic disease (Fig. 3). Due to the large size of the tumor and extensive tumor thrombosis, the multidisciplinary team overseeing this patient decided to administer neoadjuvant chemotherapy and an anticoagulant before surgical intervention. The patient was administered 12 cycles of nivolumab and cabozantinib, after which he underwent a right radical nephrectomy with an adrenalectomy and tumor thrombectomy.

**Figure 4.**
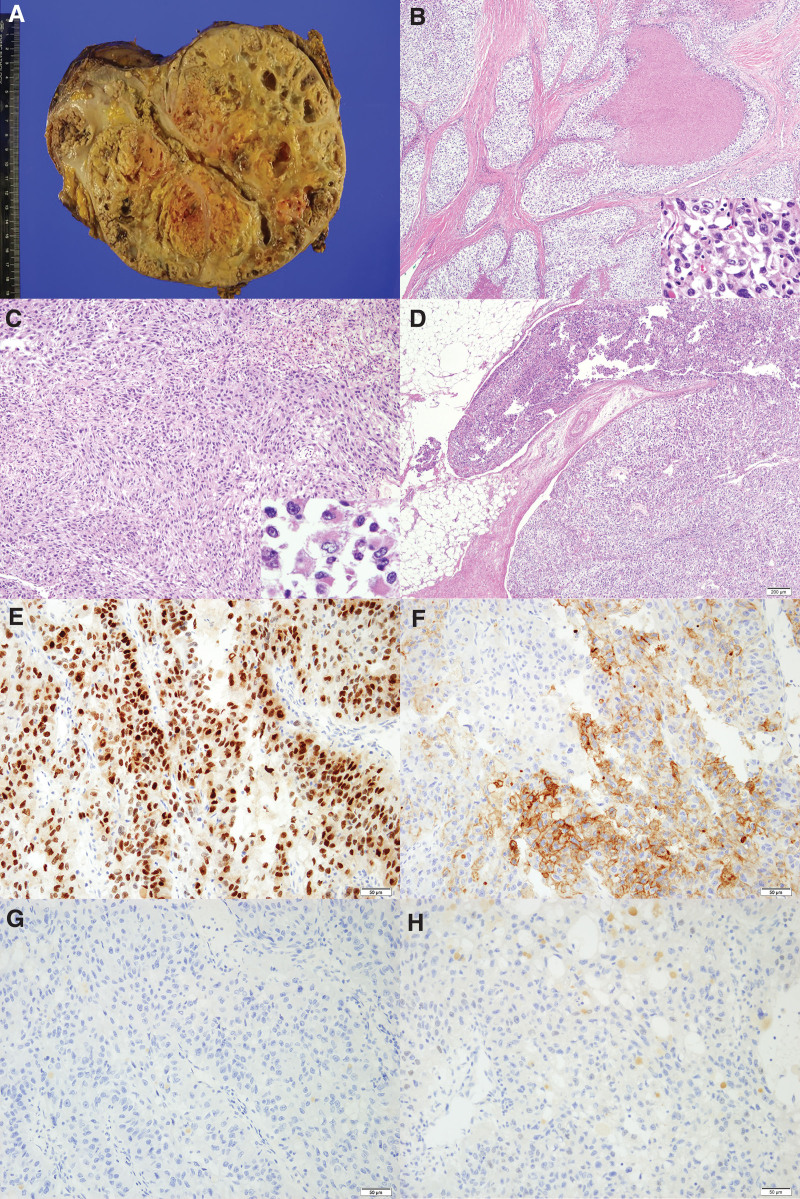
Representative gross and histological findings. (A) Gross findings, showing a huge, solid, necrotic mass in the right kidney invading the renal sinus fat and renal vein. (B) Microscopic findings, showing that the tumor consisted of large clear to eosinophilic polygonal cells forming nests with coagulative necrosis (H&E, ×40). Tumor cells had high-grade nuclear features such as pleomorphism, multinucleation, prominent nucleoli, and frequent mitosis (inset, ×400). (C) Histological findings, showing that some parts of the tumor had sarcomatoid components, consisting of pleomorphic spindle cells with fascicles arranged around vessels (×100) and focal rhabdoid cells (inset, ×400). (D) Tumor invasion of the renal sinus fat and renal vein (×40). Tumor cells were diffusely positive for PAX8 (E), patchily positive for CD10 (F), and negative for HMB45 (G) and TFE-3 (H, ×100).

Gross examination of the resected specimen showed an ill-defined, multilobulated, and exophytic mass, measuring 23.5 × 18.0 × 8.5 cm (3100 mg) throughout the entire kidney, replacing most of the renal parenchyme. The cut surface of the mass was of a tan-to-golden yellow color, with necrosis and cystic change filled with bloody clots that invaded the renal sinus fat and the wall of the vena cava (Fig. 4A). Microscopic examination showed diffuse solid sheets of polygonal cells with large nuclei and prominent nucleoli, frequent mitotic activity, and clear to eosinophilic abundant cytoplasm separated by fine delicate vasculature (Fig. 4B). In addition, 30% of the tumor consisted of sarcomatoid and rhabdoid areas, with pleomorphic spindle cells and fascicles arranged around the blood vessels (Fig. 4C). Moreover, 20% of the mass consisted of areas of coagulative necrosis that invaded the renal sinus fat and vena cava (Fig. 4D). Immunohistochemical staining showed that tumor cells were diffusely positive for PAX8 and CD10, and negative for HMB-45, TFE-3, P40, and cytokeratin-7. Further analysis showed that the sarcomatous component of the tumor was immunohistochemically positive for vimentin and the epithelial component was positive for CD10 (Fig. 4E–G). The patient was diagnosed with clear cell RCC with sarcomatoid/rhabdoid differentiation, with the tumor being of stage T3b as it did not invade the Gerota fascia. Surgery was successful, and the patient was started on a cancer rehabilitation program. The patient had no other side effects during the entire treatment process. He has been followed-up as an outpatient and no longer complains of back pain.

## 3. Discussion

Although the classic triad of RCC consists of flank pain, hematuria, and a palpable mass, these findings are present in only 10% to 14% of patients with RCC. Rather, most RCCs are asymptomatic and are usually discovered incidentally on imaging for unrelated causes.^[2]^ The present patient presented initially with simple back pain, which did not strongly indicate RCC. Imaging, however, revealed a massive RCC and a tumor thrombus. The patient was administered neoadjuvant chemotherapy and an anticoagulant, after which the tumor was removed surgically. These findings suggest that the differential diagnosis of patients who present with low back pain, even at a young age, should include the possibility of a hidden abdominal malignancy. Moreover, the results in this patient showed that large sized RCCs can be successfully treated with an appropriate approach.

The prognosis of patients with RCC depends on the stage and cytologic makeup of the tumor.^[8]^ Tumors are commonly staged using the TNM system from the American Joint Committee on Cancer staging manual, 8th edition.^[7]^ Contrast-enhanced computed-tomography is useful not only for the detection but for the staging of RCC. Although radiological imaging can noninvasively determine T category, which indicates tumor size, as well as pathological lymph nodes and distant metastases, imaging is limited in detecting the invasion of important landmarks for staging, including the renal capsule or perirenal fascia.^[9]^ Computed-tomography in the present patient showed that the RCC was 19.1 cm in size and had invaded the inferior vena cava below the diaphragm, making it of T3b or higher stage. Histopathological analysis after nephrectomy showed that the RCC was 23.5 × 18.0 × 8.5 cm in size, but did not invade the Gerota fascia, confirming that it was a stage T3b tumor.

Histological factors prognostic of tumor outcomes in patients with RCC include tumor morphology, sarcomatous/rhabdoid differentiation, and tumor necrosis.^[10]^ Pathologic slide reviews showed that clear cell RCC presents at a higher pathologic stage, has a higher risk of metastasis, and therefore has lower cancer-specific survival than papillary or chromophobe RCC.^[11,12]^ Sarcomatous/rhabdoid differentiation is a form of dedifferentiation, associated with advanced stage with metastasis and poor patient prognosis. Patients with clear cell RCC have a 5-year cancer-specific survival rate of 15% to 22% and an average survival period of 4 to 9 months, whereas patients with papillary or chromophobe RCC have a cancer-specific mortality rate of 40% to 50% and an average survival period of 8 to 31 months. RCCs with sarcomatous and/or rhabdoid differentiation correspond to grade 4 on the World Health Organization/International Society of Urological Pathology grading system.^[13]^

Tumor necrosis has also been associated with poor patient prognosis. The International Society of Urological Pathology consensus recommended that the presence and rate of necrosis be included in the pathology report. A grading system that included the presence or absence of necrosis was found to allow greater prediction of outcomes,^[10]^ including disease-free survival and cancer-specific survival.^[14]^ Histopathologic analysis of the tumor removed from the present patient showed that it was a clear cell RCC,^[15,16]^ with 30% of the mass consisting of sarcomatous and rhabdoid undifferentiated areas and 20% consisting of areas of coagulative necrosis. These findings were consistent with a grade 4 tumor on the World Health Organization/International Society of Urological Pathology grading system, with the tumor expected to have a high risk of metastasis and to be associated with a low patient survival rate. Because 30% of these patients develop metastatic disease after complete resection of the primary tumor,^[17]^ this patient was expected to have a high probability of developing metastasis, therefore requiring frequent follow-up.

Although surgery is generally the first-line treatment for RCC, a giant RCC accompanied by a tumor thrombus, as in this patient, may be inoperable. Medical treatment may be an alternative, with immune checkpoint inhibitors being approved as frontline treatment for patients who were initially inoperable and had a high tumor burden. Multiple cycles of treatment with immune checkpoint inhibitors reduce the size of the primary tumor, allowing subsequent tumor resection.^[18]^

## 4. Conclusions

In conclusion, this case report showed RCCs can manifest clinically as back pain, and that appropriate imaging modalities may enhance diagnosis. RCC should be considered in the differential diagnosis of patients, even younger patients, who present with low back pain.

## Author contributions

**Conceptualization:** Ga Yang Shim, Myung Chul Yoo.

**Data curation:** Hong Jun Kim, Min-Su Kim, Yewan Park, So-Woon Kim.

**Formal analysis:** Ga Yang Shim, Myung Chul Yoo.

**Investigation:** Ga Yang Shim, Myung Chul Yoo.

**Methodology:** Ga Yang Shim, So-Woon Kim, Myung Chul Yoo.

**Supervision:** Myung Chul Yoo.

**Validation:** Myung Chul Yoo.

**Writing – original draft:** Ga Yang Shim, Min-Su Kim.

**Writing – review & editing:** Myung Chul Yoo.
